# *Trans*-regulation of RNA-binding protein motifs by microRNA

**DOI:** 10.3389/fgene.2014.00079

**Published:** 2014-04-15

**Authors:** Francis Doyle, Scott A. Tenenbaum

**Affiliations:** Nanobioscience Constellation, College of Nanoscale Science and Engineering, State University of New YorkAlbany, NY, USA

**Keywords:** post-transcriptional regulation, dark matter, RNA-binding proteins (RBPs), microRNA (miRNA), non-coding RNA, stem-loop binding protein (SLBP), structural interacting RNA (sxRNA)

## Abstract

The wide array of vital functions that RNA performs is dependent on its ability to dynamically fold into different structures in response to intracellular and extracellular changes. RNA-binding proteins regulate much of this activity by targeting specific RNA structures or motifs. One of these structures, the 3-way RNA junction, is characteristically found in ribosomal RNA and results from the RNA folding in *cis*, to produce three separate helices that meet around a central unpaired region. Here we demonstrate that 3-way junctions can also form in *trans* as a result of the binding of microRNAs in an unconventional manner with mRNA by splinting two non-contiguous regions together. This may be used to reinforce the base of a stem-loop motif being targeted by an RNA-binding protein. *Trans* interactions between non-coding RNA and mRNA may be used to control the post-transcriptional regulatory code and suggests a possible role for some of the recently described transcripts of unknown function expressed from the human genome.

## Introduction

RNA performs a broad variety of fundamental cellular functions, many of which depend on its ability to actively fold into numerous conformational structures. These RNA structures can form motifs, which are frequently bound by an assortment of RNA-binding proteins (RBPs). In eukaryotic organisms, this provides a means to couple transcription with various levels of post-transcriptional gene regulation such as splicing, nuclear export, RNA localization, turnover, and translation (Dreyfuss et al., 2002; Keene, 2007; Dinger et al., 2011; Ascano et al., 2013; Mercer and Mattick, 2013). Researchers now regularly study these interactions by immunoprecipitating RNA-protein complexes (RNPs) and identifying the associated RNA cargo using genomic readouts including microarrays and more recently next-generation sequencing. These techniques are frequently referred to as RIP-Chip, PAR-CLIP and RIP-Seq (Tenenbaum et al., 2000; Brown et al., 2001; Intine et al., 2003; De Silanes et al., 2004; Gerber et al., 2004, 2006; Di Marco et al., 2005; Hogan et al., 2008; Mazan-Mamczarz et al., 2008; Calaluce et al., 2010; Abdelmohsen et al., 2012; Guttman and Rinn, 2012; LeGendre et al., 2013; Singh et al., 2014). These methods have proven to be powerful tools for studying the role of RBPs in post-transcriptional gene expression and for subtly dissecting this complex process to uncover the players involved. The resultant data has led to the proposal that a combinatorial code allows for the regulation of multi-functional genes producing a tremendously complex number of recipes, from a limited number of genetic ingredients (Keene and Tenenbaum, 2002). Exactly how this dynamic, post-transcriptional regulatory code is modulated so that multi-functional ingredients are produced correctly for each of the various recipes they are used in has remained elusive. The same can be said for alternatively spliced genes.

## *Trans*-interactions of non-coding RNA with mRNA

In our experience and depending on the RBP being targeted, it is not uncommon to detect non-coding RNAs (ncRNA) as part of a RIP. This has become increasingly true as more comprehensive genomic tools, such as tiling-arrays and next generation sequencing technologies have been used. Although experimentally challenging to definitively prove, at least a portion of this ncRNA appears to be *indirectly* associated with the mRNP complex, resulting from binding to the mRNA cargo and not directly to the RBP. Much of this ncRNA is uncharacterized but some are microRNAs (miRNAs). Conventional models of miRNA regulation are based on perfect or imperfect linear hybridization to mRNAs, which results in the targeted mRNA being degraded and/or inhibited at the translation level. The former's mechanism is well explained by direct cleavage of the mRNA, however the latter's is not yet perfectly elucidated. It is possible that in searching for a mechanism similar to the originally identified destructive pathway, scope was artificially limited to cases with reduced translation of targeted mRNAs. However, as an mRNA cannot be “slightly cleaved,” it is logical to assume that repressive effects of imperfect miRNA hybridization result from a wholly separate, second process rather than simply an attenuated version of the first. If the second process is more generic, it could conceivably manage multiple downstream responses, perhaps via affecting third party elements such as RBPs. In fact, examples of increased translation of a miRNA targeted mRNA have been reported (Vasudevan et al., 2007; Srikantan et al., 2011), as have instances of viruses that utilize endogenous cell miRNA in “unconventional” manners to promote their own replication (Machlin et al., 2011).

## Structural implications of trans-acting miRNA

Some miRNAs may target an overlapping regulatory code with RBP motifs and, in addition to the accepted functions for miRNA, may also influence the structure of mRNA and subsequently, the availability of potential RBP motifs (George and Tenenbaum, 2006). When RBPs interact with their mRNA cargo they frequently bind to motifs that contain one or more stem-loop structure that are assumed to form in *cis* within the message (Grillo et al., 2010; Thapar et al., 2014). Specifically, we suggest that miRNAs can bind mRNA in manner that influences the local structure of the associated mRNA, subsequently influencing the availability or affinity of RBP binding sites (or vice versa). These “*trans”* miRNA-mRNA interactions could impact the structural potential, context, and accessibility of multiple RBP binding sites simultaneously and/or sequentially and more importantly, in a dynamic and combinatorial manner. *Trans*-ncRNA interactions of this nature would have the potential for adding tremendous complexity and a hierarchy to the post-transcriptional regulatory code. They would also provide a possible function for at least a portion of what has been referred to by some as the “Dark Matter” expressed from many genomes (Kapranov et al., 2002, 2007a,b, 2010; Mattick, 2003; Kapranov and St. Laurent, 2012; Clark et al., 2013). Modulation of structure is a simple, yet elegant system that would provide miRNA (as well as other ncRNA) the potential for a vast range of control including, but not limited to, its current characteristic role as an inhibitor.

## *Trans* three-way junctions

**Figure 1:**
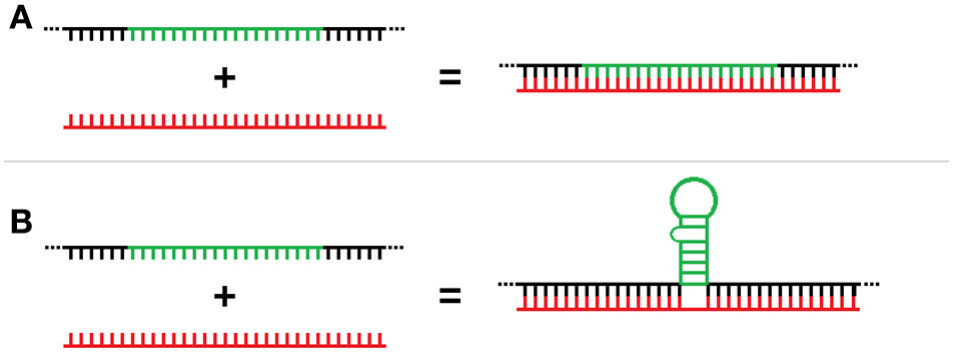
**Trans-acting 3-way junction model. (A)** Representation of traditional miRNA-mRNA double stranded duplex formation as compared to **(B)** the proposed trans 3-way junction sxRNA structure, which would result from the splinting of a miRNA discontinuously across the flank region of the mRNA located at the base of a stem-loop element.

Three-way RNA junctions (3WJs) are common subdomains critical to the function of numerous structured RNAs (e.g., group I introns, rRNA, etc) and form when the RNA folds to produce three separate helices meeting around a central unpaired region (Lescoute and Westhof, 2006). However, 3WJs could also form as a result of the unconventional binding of miRNAs to the base of a stem-loop that can also act as RBP target motifs. Unlike traditional miRNA-mRNA double stranded duplex formation (Figure 1A), these *trans* 3-way junctions would result from the splinting of the miRNA *discontinuously* across the flank region of the mRNA located at the base of the stem-loop element (Figure 1B). These 3WJs are structurally similar to those well-established in rRNA but, unlike the *cis* versions formed in rRNA, these 3WJs are formed in *trans* from two independent RNAs. This interaction represents a potentially new post-transcriptional regulatory role for ncRNA and we have termed these interactions “*structural interacting* RNAs” or sxRNA for short.

## The post-transcriptional regulation of histone genes

Many RBPs interact with their mRNA cargo by binding to motifs containing one or more stem-loop structure (Grillo et al., 2010; Thapar et al., 2014). A prototypic example of this is the histone stem-loop (HSL) binding protein (SLBP), which binds to the HSL (Dominski and Marzluff, 1999; Marzluff and Duronio, 2002; Zhang et al., 2012). Histone proteins play a major role in regulating transcriptional activity and gene expression by packaging DNA into chromatin (Jenuwein and Allis, 2001). There are five major families of histones: H1/H5, H2A, H2B, H3, and H4 and their mRNAs are unique in that they are the only naturally occurring eukaryotic mRNAs that often lack introns and that are not typically polyadenylated (Marzluff et al., 2008). Instead the regulation of metazoan histones requires a specialized post-transcriptional mechanism using the conserved HSL motif comprised of a 16-base stem-loop structure with additional flanking sequence. This motif is found at the extreme 3′-end of most metazoan histone mRNAs and is targeted by SLBP (Dominski and Marzluff, 1999; Marzluff and Duronio, 2002; Zhang et al., 2012). When bound, SLBP greatly increases the translation of the associated mRNA, in some cases by an order of magnitude or more (Dominski and Marzluff, 1999; Marzluff and Duronio, 2002; Zhang et al., 2012). The HSL is essential to histone pre-mRNA transcription termination as well as mature mRNA nuclear export, translation efficiency and stability (Dominski and Marzluff, 1999; Marzluff and Duronio, 2002; Marzluff et al., 2008; Zhang et al., 2012). It also provides a way to coordinate expression of histone genes in a manner that is tightly synchronized with cell cycle, which is essential for the unique role of histones (Dominski and Marzluff, 1999; Marzluff and Duronio, 2002).

What, if any effect the Argonaut or RISC proteins would have on the potential of sxRNA complexes to form or what their biological function may be is difficult to predict and if they occur could be transient and difficult to characterize. Though not definitive proof, PAR-CLIP data for Argonaut proteins, including AGO2, exists (Hafner et al., 2010; Kishore et al., 2011) that is suggestive of an sxRNA-like foot-printing of HSL-structural motifs of 40–60 bases in some histone genes, which is consistent with the sxRNA interactions we have informatically predicted as opposed to traditional miRNA-mRNA foot-printing, which would be shorter and lacking structure.

## miRNA-HSL 3WJs

**Figure 2:**
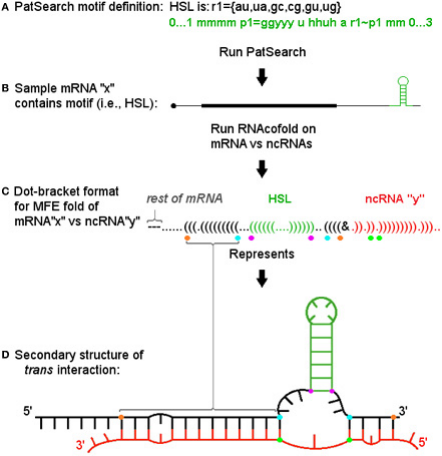
**Informatic approach and pipeline for identifying trans 3-way junctions. (A)** The PatSearch program is used to identify a subset of mRNAs from Refseq that contain a defined motif. Motif location within each mRNA is retained. **(B)** Each motif (shown in green) containing mRNA (here a single representative, “x,” is shown) is run through RNAcofold vs. each ncRNA in query set (e.g., “mirBase”) for both whole message as well as a ±25 b sequence window surrounding motif. **(C)** Each resulting minimum free energy (MFE) fold's (here a single example fold with ncRNA, “y,” is shown) dot-bracket output was parsed using the known location of the motif to i. ascertain if motif structure is present (again, in green), ii. determine if the ncRNA (shown in red) hybridizes across the motif's base, iii. then, if both these conditions are met, to identify positions defining critical segments for scoring the sxRNA. These positions are denoted by colored dots below corresponding dot-bracket characters. Purple dots denote bottom bases of motif stem. Blue dots denote first mRNA base hybridized to ncRNA 5′ and 3′ of the motif. Orange dots denote last mRNA bases hybridized to ncRNA 5′ and 3′ of motif (where “first” and “last” are relative to distance from motif base). The green dots denote the ncRNA bases paired with the blue dot mRNA bases. **(D)** The secondary structure described by the dot-bracket notation is shown. Colored dots identified in previous section are shown at their corresponding base positions. The segments they define can be easily identified in this format. Segments between purple and blue dots define the mRNA side junction regions. Segment between green dots defines the ncRNA side junction region. Segments between blue and orange dots define extent of 5′ and 3′ helices on mRNA side.

To find potential examples of our model, we used the well-characterized HSL motif and determined the potential for miRNAs to target this structure and form a 3WJ. We first identified the set of HSL containing human histone mRNAs present in the Refseq hg19 annotation by using the PatSearch program (Grillo et al., 2003). We then identified potential *trans*-3WJs using predictions based on the well-established descriptions for *cis*-3WJs (Lescoute and Westhof, 2006). Figure 2 depicts the general approach we used (with dot-bracket style output representing the related secondary structure) and illustrates the minimum free energy fold as parsed and analyzed by a Java program to determine if: (1) the motif structure is present in the cofold; (2) the ncRNA binds across the motif's base and; (3) the resultant interaction meets thresholds based on guidelines for *cis* 3-way junctions (e.g., number of non-paired bases in junction regions) and for general hybridization characteristics (e.g., percentage of paired bases in miRNA and HSL flank regions).

**Figure 3:**
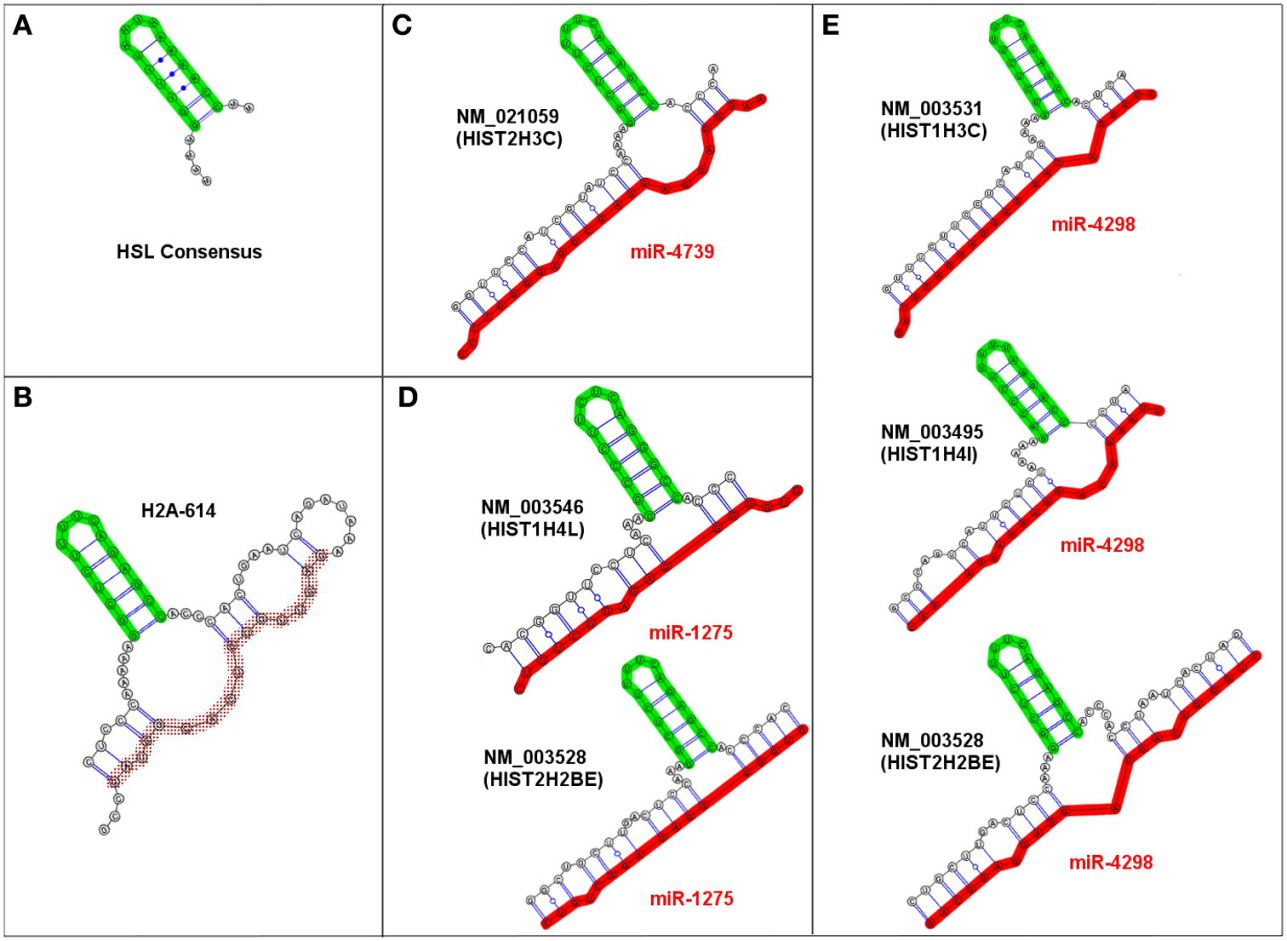
**Examples of predicted HSL-sxRNA structures depicted using RNAcofold. (A)** The consensus HSL. **(B)** The pre-processed HSL sequence containing the histone downstream element sequence predicted to form a cis-three way junction structure that is similar to the trans sxRNA (Zhang et al., 2012). **(C–E)** Representative predicted HSL-sxRNA structures for miR-4739, miR-1275, and miR-4298. Three representative miRNAs (miR-4739, miR-1275, and miR-4298) potentially interact with multiple histone mRNAs, all of which form a conspicuously similar potential 3-way junction sxRNA structure. Note that in every observed instance, the adenosines at the 5′ base of the stem-loop (depicted to the left of the base of the HSL motif) are consistently predicted to form a non-base paired bulge at the base of the HSL. These adenosines are vital for proper SLBP binding and were always predicted to be unpaired.

We identified multiple examples of very similar *trans*-3WJs structures that were predicted to form irrespective of the specific miRNA-mRNA combination and fit our criteria for an sxRNA. Figure 3 depicts examples of these sxRNAs illustrated using RNAcofold (Lorenz et al., 2011). In some cases, the same miRNA was observed to associate with more than one histone mRNA and as depicted in Figures 3D,E, three of the miRNAs (miR-4739, miR-1275, and miR-4298) were predicted to interact with six different histone mRNAs, all of which form a conspicuously similar structure. Strikingly, in every observed instance, the adenosines at the 5′ base of the stem-loop are always predicted to form a non-base paired bulge (depicted to the immediate left of the base of the HSL motif), which may be needed to properly orient the stem-loop in space. These adenosines have been shown to be essential for proper SLBP binding and may need to remain unpaired (Marzluff and Duronio, 2002; Zhang et al., 2012).

**Figure 4:**
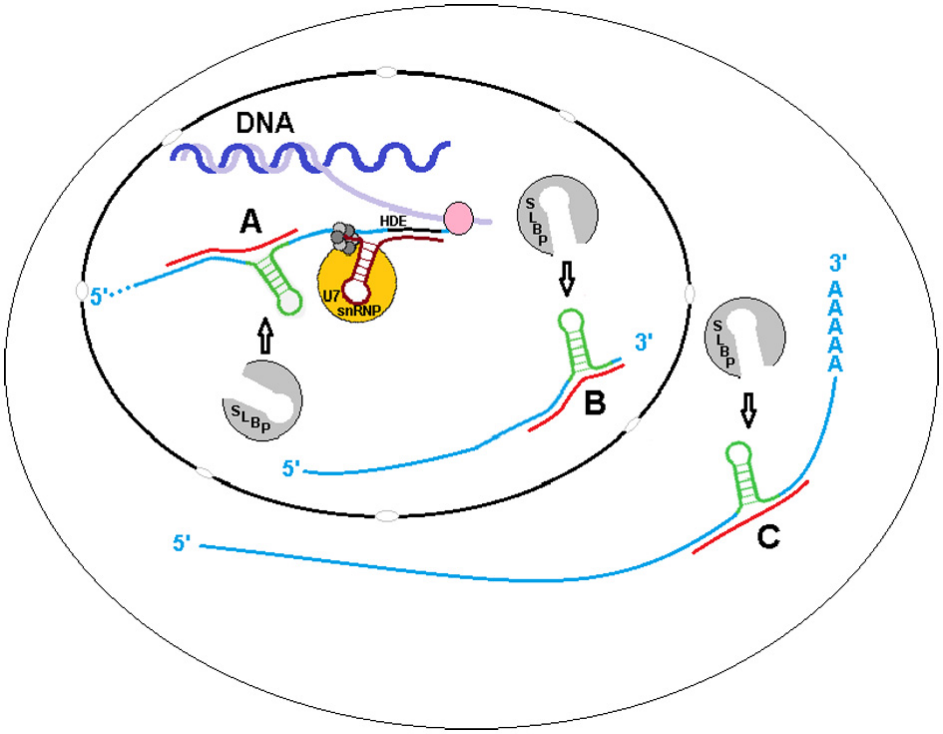
**sxRNA in Histone Metabolism.** The binding of the Histone Stem Loop (HSL) found at the 3′ terminal end of metazoan histone mRNAs by Stem Loop Binding Protein (SLBP) is known to affect their transcription end processing, nuclear export and enhance translation in lieu of a poly(A) tail. Stabilization of the HSL by a ncRNA formed trans 3-way junction may occur during **(A)** transcription or, as it is believed that SLBP involved in transcription termination does not remain associated with the mRNA (Sullivan et al., 2009) to **(B)** promote re-association of the message with SLBP for export. After this stage, cytoplasmic histone messages are found trimmed to ~2 bases 3′ of the stem, removing the flanking sequence required for 3-way junction formation, with the possible exception of atypical polyadenylated transcripts with internalized HSL motifs. A potential interaction with one of these variants **(C)** in the cytoplasm is depicted.

Although we have determined that sxRNA complexes can form artificially (data not shown), it is yet to be shown that these HSL-sxRNAs naturally form or if they play a functional role in histone metabolism. However, histone metabolism is a very elegant and tightly regulated essential cellular process that has many layers, any of which could use the type of post-transcriptional regulation we have predicted (Figure 4). For example, the HSL-SLBP interaction is known to affect histone mRNA transcription end processing, nuclear export and enhance translation in lieu of a poly-A tail. Stabilization of the HSL by a miRNA-induced 3WJ could occur during transcription or transcriptional termination or to promote re-association of the histone message with SLBP for export. After this stage, cytoplasmic histone messages are trimmed to ~2 bases 3′ of the stem, which would remove the flanking sequence necessary to form the predicted 3WJ. However, the exonuclease responsible for this trimming must first bind to the HSL cooperatively with, and promoted by, previously bound SLBP (Tan et al., 2013). Therefore, if HSL-sxRNA stabilization occurs naturally, it would likely occur in the nucleus. A possible exception might be found in the atypical histone transcripts that have both HSL and poly(A) terminated variants, the latter including an upstream HSL sequence. It is possible that *trans* stabilization of this internalized HSL plays some role in the cytoplasm (Figure 4C).

## Other examples of sxRNA

**Figure 5:**
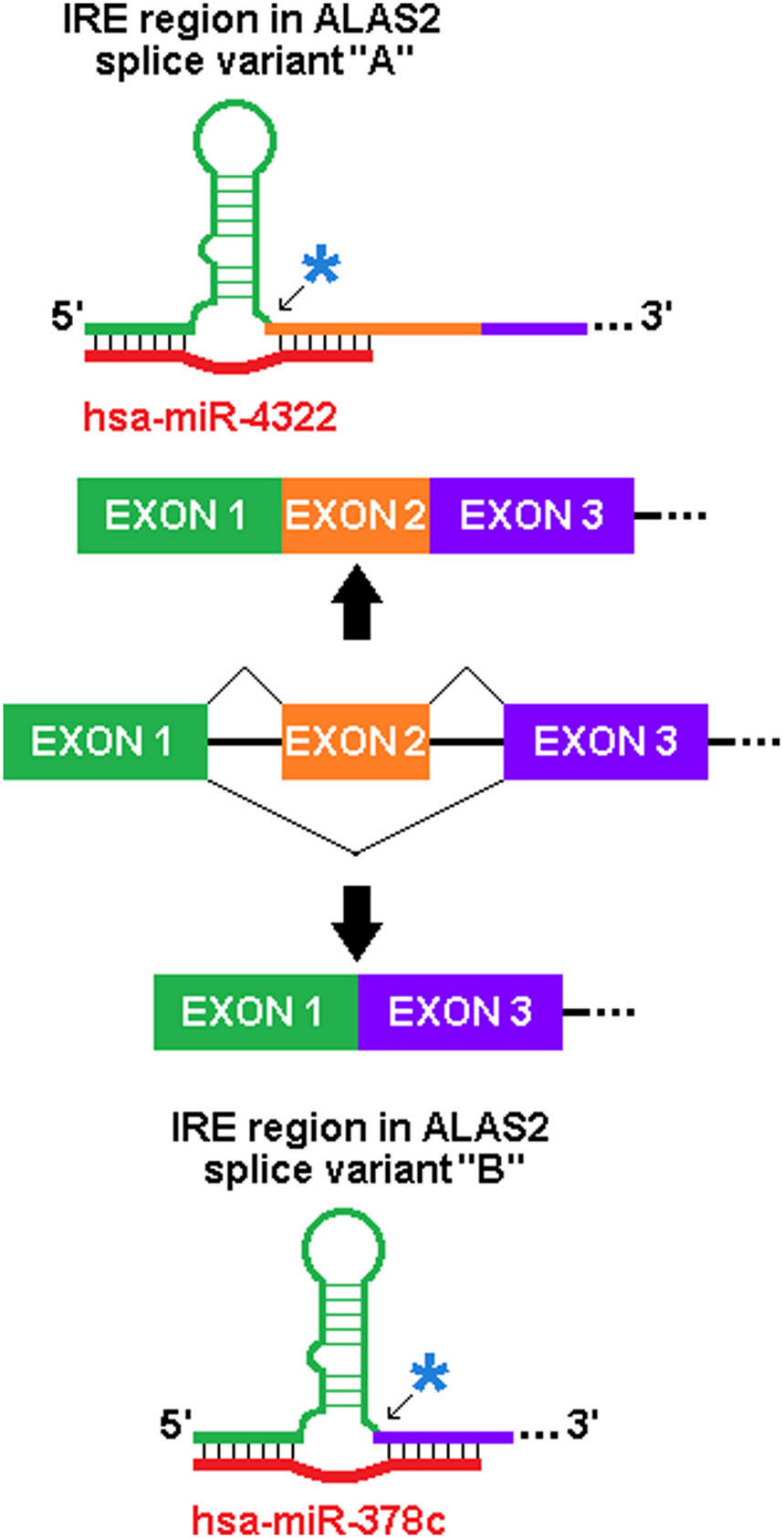
**Potential of sxRNA Targeting to Affect Alternative Splicing.** The incorporation of an alternatively spliced exon immediately 3′ of the base of the IRE stem (noted by blue asterisks and arrows) in ALAS2 mRNA transcript variants alters which miRNA is predicted to interact (variant “A” uses RefSeq Accession NM_001037968 and “B” uses NM_001037967). This particular sxRNA interaction suggests a possible influence on the alternative splicing process and may be indicative of sxRNA post-transcriptional modulators being coupled to transcriptional regulation. Note: The IRE, and genomic exons and introns are not depicted to scale.

We have identified similarly predicted sxRNAs for the iron-response element (IRE), which is the target of the IRE-binding proteins that regulate the post-transcriptional expression of genes involved in iron metabolism. A dramatic example of this is found in the erythroid-specific ALA synthase gene (ALAS2), a critical protein whose misregulated activity is associated with both sideroblastic anaemia (May and Bishop, 1998) and protoporphyria (Whatley et al., 2008). As represented in Figure 5, this gene has multiple isoforms that contain different IRE 3′ flanks formed by the alternative splicing of different exons, as the IRE is immediately proximal to the splice junction. Two different miRNA (mir-378c or miR-4322) were predicted to form distinct sxRNAs with each of these variants. This strongly suggests a possible sxRNA role related to the alternative splicing of this gene.

## Competing sxRNAs

**Figure 6:**
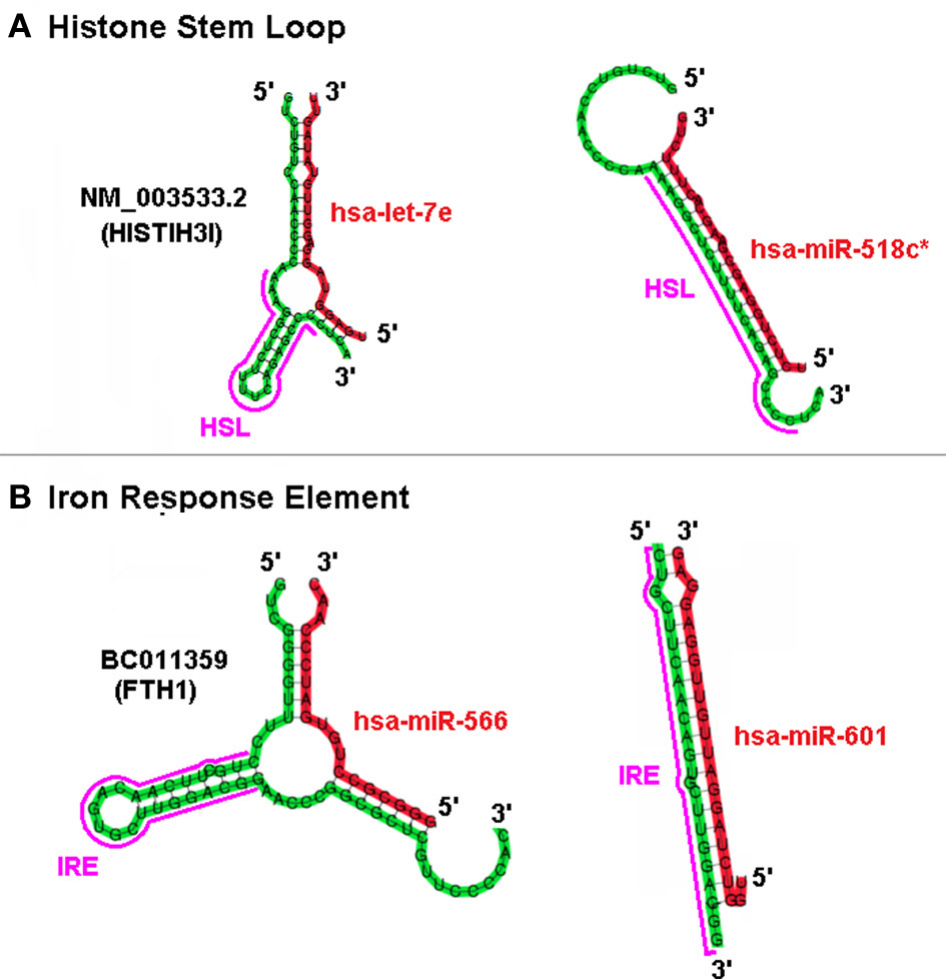
**Positive and Negative sxRNA Interactions on the HSL or IRE Motif.** The UTR portion of two mRNA is depicted folded using co-Fold. Shown are, **(A)** NM_003533 (HIST1H3I) histone message and **(B)** BC011359 ferritin, heavy polypeptide 1. Each mRNA has a miRNA that either induces/strengthens the target stem loop RBP binding-site motif (HSL or IRE) or ablates it.

Based on the predicted structure of a putative miRNA interaction at a site it is plausible that “positive” sxRNAs with potential “negative” miRNA counterparts could compete for the same region of a target mRNA but with opposing outcomes. For example, the top panel in Figure 6 depicts the miRNA hsa-let-7e acting as an inducer sxRNA and hsa-miR-518c∗ acting as a repressor sxRNA for the same HSL sequence in the 3′ UTR of the HIST1H3I histone message. A fundamental difference between potential positive and negative interactions is that each of the negative miRNA can target many histone mRNAs because they complement the conserved histone stem-loop sequence rather than the non-conserved flank regions of the stem-loop. A similar observation was made for miR-566 and miR-601 that target the same IRE stem-loop structure located in the UTR of the heavy polypeptide-1 ferritin mRNA (Figure 6 bottom panel). Using mixtures of competing sxRNAs, nature could use ncRNA in a combinatorial manner to mask and/or reveal various potential regulatory elements located in the mRNA of a multi-functional gene.

## Discussion

Unlike DNA, RNA has the tremendous potential to form complex and elegant structures (such as aptamers) and this may be the single most biologically important attribute of RNA. This property permits RNA to serve both as an intermediate for conveying the genetic code while also creating unique surfaces for performing protein-like enzymatic functions in the cell. This also allows mRNA to simultaneously encode for a protein as well as regulate where, when and how much of the mRNA will be translated into this protein. Accordingly, eukaryotic genes are not exclusively transcriptionally regulated, but are also highly regulated at the post-transcriptional level, typically mediated by RBPs, which have repeatedly been shown to facilitate the expression of different mRNA isoforms, localize specific mRNAs and influence variable mRNA translation(Lunde et al., 2007; Mittal et al., 2009). Many of these RBPs perform their function in a manner dependent on both sequence and specific structural context and conformations present in their target RNA motifs, which often contain stem-loop structural components residing in the UTRs of the mRNA (Brown et al., 2001; De Silanes et al., 2004; Lunde et al., 2007; Mittal et al., 2009; Mukherjee et al., 2011). Accordingly, the association of specific RBPs can ultimately determine the splicing pattern, expression level or even cellular localization/sequestration of a particular mRNA.

In recent years, large numbers of transcribed ncRNA from the human genome have been detected (sometimes referred to as “transcriptional noise” from genomic “junk” regions), and it is reasonable to assume that at least some will be found to play a regulatory role (Kapranov et al., 2002, 2007a,b, 2010; Dunham et al., 2012; Kapranov and St. Laurent, 2012). These include piRNA, lncRNA, and new flavors of miRNA. Research into small ncRNAs and more specifically miRNAs, has also brought to light new layers of post-transcriptional gene-expression regulation. Conventionally accepted as repressors, ample evidence exists that miRNAs may also behave as activators of translation, though the mechanism for this behavior is not fully understood (Pawlicki and Steitz, 2008; Abdelmohsen et al., 2011; Carroll et al., 2012; Woo et al., 2013). Multiple studies have also shown that the binding sites for many miRNAs reside specifically in the 3′UTRs of target mRNAs and may be influenced by and/or be influencing RBP activity(Bhattacharyya et al., 2006; George and Tenenbaum, 2006; Galgano et al., 2008; Kim et al., 2009; Jacobsen et al., 2010). Here we have proposed a new type of miRNA interaction, one that has the tremendous potential to expand the post-transcriptional regulatory capacity by influencing the conformational landscape of mRNA and thus the regulatory elements contained therein.

Histone proteins play an essential role in packaging DNA into chromatin and in regulating the “histone code” of gene expression. In humans, there are approximately 60 identified histone genes that code for the five core histone proteins H1, H2A, H2B, H3, and H4 (Marzluff et al., 2008). Yet for any given core family, most of these genes are identical (or virtually identical) at the protein sequence, frequently only showing differences at the nucleotide level, and then predominantly in the 3′-UTR (Jenuwein and Allis, 2001). Our model suggests that this diversity may be functional and allow for fine-tuning of specific histone gene expression at the post-transcriptional level. This could provide for elegant localized translation of specific histone protein isoforms to facilitate the regulatory capacity of the “histone code” and its role in transcriptional control.

Although we have concentrated on the SLBP and HSL as the focus of the present study, we anticipate that a broader network of miRNA and other ncRNA likely exists that could influence the structure of other RNA by acting in *trans*. Our sxRNA hypothesis predicts that some miRNAs modulate RBP-binding sites in a dynamic manner and target a shared regulatory code in the UTR region of mRNAs. Further, sxRNA provides a mechanism for *trans*-acting ncRNA to influence the structure or shape of RBP regulatory elements on an mRNA thereby influencing the optimal expression of a multi-functional mRNA transcript. Mixtures of potential sxRNA interactions can be envisioned in which the miRNA indirectly or directly influences an existing RBP structural element. This could be performed in either a negative (repressor) manner, by eliminating the RBP binding motif, or a positive (inducer) mode by assisting an RBP in binding. Additionally, the miRNA could even help form the RBP motif by actually comprising a portion of it (see Figure 6). These types of sxRNA interactions could influence the affinity/binding of an RBP for its regulatory element and provide either a discrete “ON-OFF” or even variable amplitude type switching mechanism for regulation, similar to the memory versus amplifier role for transistors in electronic circuits.

## A model for trans RNA modulation of RBP motifs

**Figure 7:**
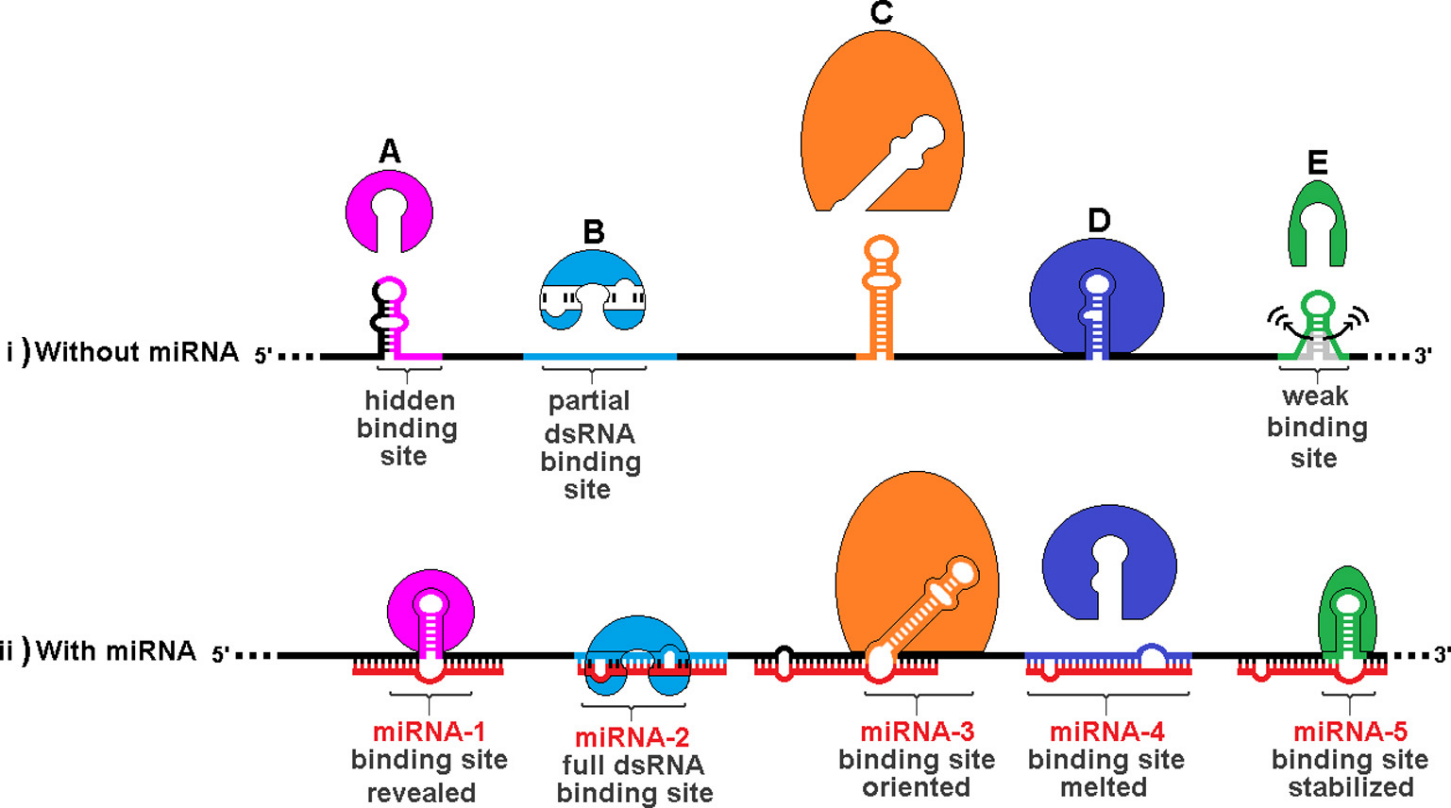
**Trans RNA modulation of RNA Binding Protein (RBP) structural binding sites.** The same hypothetical mRNA is shown in both (i) without associated miRNAs and (ii) with these miRNAs. A number of different functional mechanisms for trans-acting modulation are depicted: **(A)** An RBP whose binding site is hidden by an alternate structure. In the presence of the associated trans-acting ncRNA, the site is revealed. **(B)** An RBP that recognizes a double stranded binding site that only forms via interaction with a separate ncRNA. **(C)** An RBP whose binding site must be presented in a particular orientation, with respect to its flanking sequence, that is facilitated by a 3WJ. **(D)** An RBP whose binding site is hidden by its sequence hybridizing with an ncRNA. **(E)** An RBP whose binding site structure is weak and may sample alternate structures over time. A trans-acting 3WJ stabilizes the active conformation, allowing the protein to bind. This combined model allows a single mRNA transcript variant to exhibit multiple behaviors amongst cell types via the combinatorial absence and presence (in varying concentrations) of both the relevant RBPs and, more specifically, ncRNAs. It is these latter that may facilitate a distinct regulation for sub-populations of expressed mRNAs, where a much broader group may hold the potential for control by the same RBP.

Figure 7 illustrates the varieties of sxRNA we have envisioned and depicts a hypothetical composite mRNA in the presence or absence of any hypothetical associated miRNAs. A number of different functional mechanisms for trans-acting modulation are represented including: (**A**) An RBP whose binding site is hidden by an alternate structure. In the presence of the associated trans-acting ncRNA, the site is revealed. (**B**) An RBP that recognizes a double stranded binding site that only forms via interaction with a separate ncRNA. (**C**) An RBP whose binding site must be presented in a particular orientation, with respect to its flanking sequence, that is facilitated by a 3WJ. (**D**) An RBP whose binding site is hidden by its sequence hybridizing with an ncRNA. (**E**) An RBP whose binding site structure is weak and may sample alternate structures over time and an sxRNA stabilizes the active conformation, allowing the protein to bind. This model allows a single mRNA transcript variant to exhibit multiple behaviors amongst cell types via the combinatorial absence and presence (in varying concentrations) of both the relevant RBPs and, more specifically, ncRNAs. It is these latter that may facilitate a distinct regulation for sub-populations of expressed mRNAs, where a much broader group may hold the *potential* for control by the same RBP.

**Figure 8:**
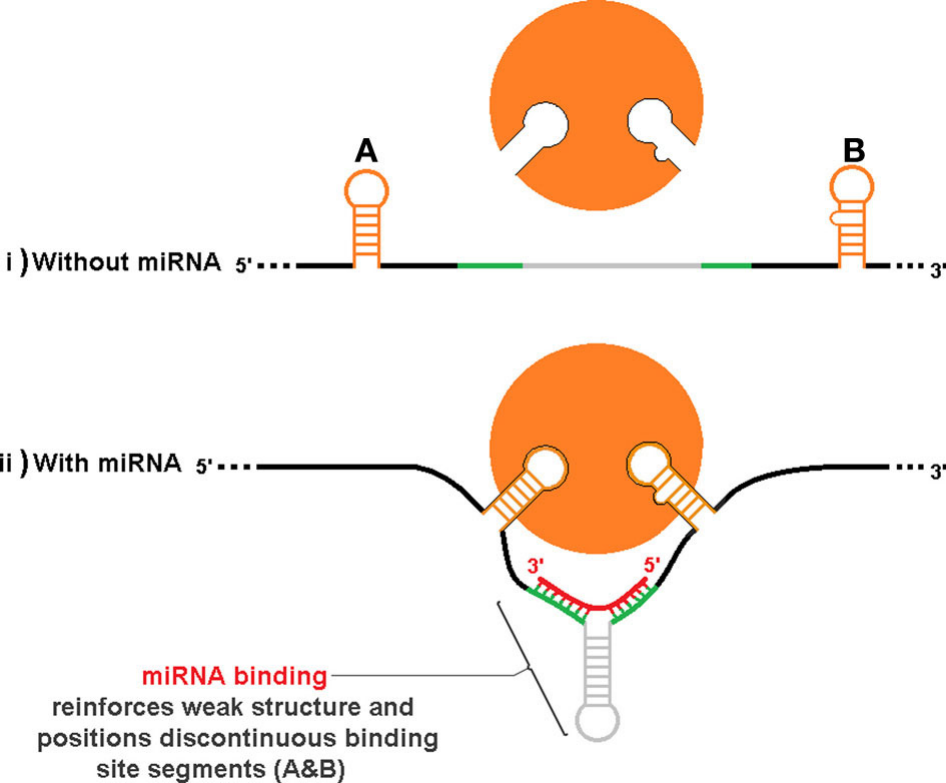
**Indirect influence of trans RNA modulation on complex RBP structural binding sites.** As in Figure 7, the same hypothetical mRNA is shown in both (i) and (ii), without and with an associate miRNA, respectively. Binding of the miRNA strengthens or orients a structure such that discontinuous RBP binding motif segments (**A,B**) are brought into proximity and/or orientation to facilitate binding to the protein. This is an abstract schematic, and is meant to represent a variety of possible scenarios where local structure may affect regional conformation such as to inhibit or promote function.

An additional level of sxRNA regulation could occur by indirectly influencing tertiary structure to reveal (or hide) complex regulatory motifs that must be properly oriented in space. Figure 8, depicts a hypothetical stretch of mRNA with or without an associated miRNA that when forming an sxRNA strengthens or orients a structure such that discontinuous RBP binding motif segments (Figures 8A,B) are now brought into optimum proximity and/or orientation to facilitate binding of an RBP. This is an abstract schematic, and is meant to represent a variety of possible scenarios where local structure may affect regional conformation such as to inhibit or promote function.

## Biological consequences of sxRNA

**Figure 9:**
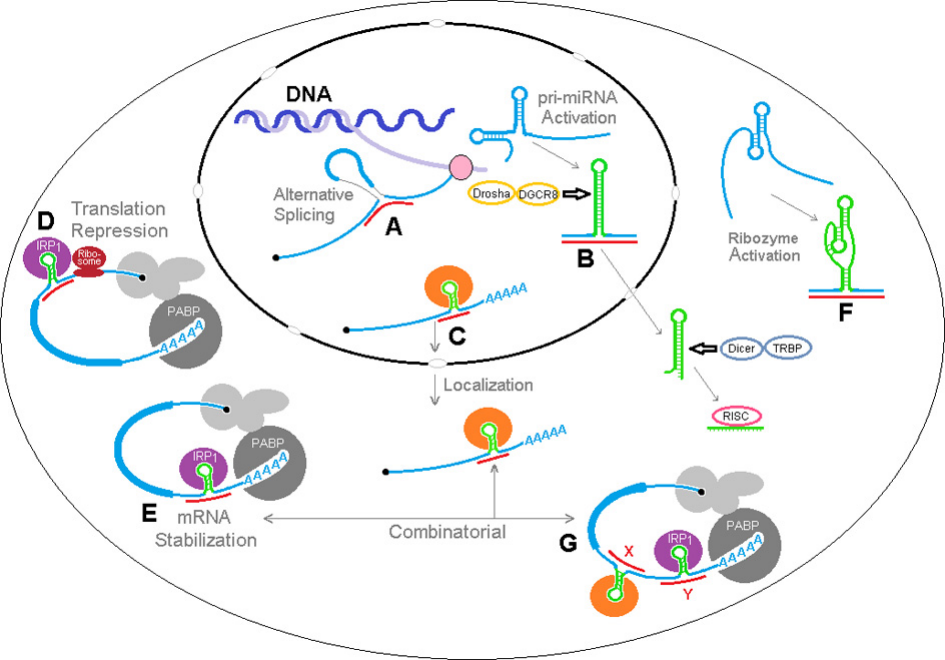
**sxRNA model of RNA structural dynamics and potential post-transcriptional regulation.** sxRNA There are a multitude of downstream results that may be modulated by a ncRNA to mRNA 3-way junction. A number of hypothetical effects are depicted where ncRNAs could: **(A)** interact with nascent pre-mRNA to affect alternative splicing, perhaps by affecting splicing factor recognition sites or (as depicted here) by binding to exon regions on both sides of a particular splice junction, **(B)** affect the structural conformation of an miRNA primary transcript, determining its availability for trimming to pre-miRNA and nuclear export, **(C)** reinforce a stem loop binding motif for a localization RBP, determining mRNA shuttling destination, **(D)** further lessen translation via iron regulatory protein by stabilizing a 5′ IRE in particular mRNAs, **(E)** alternatively, enhance mRNA stabilization via iron regulatory protein by stabilizing a 3′ IRE in particular mRNAs **(F)** activate structure dependent ribozymes, **(G)** act in a combinatorial fashion with multiple signals in a single mRNA.

We have envisioned a number of hypothetical biological effects of sxRNA regulation, which are depicted in Figure 9 where miRNAs could: (**A**) interact with nascent pre-mRNA to affect alternative splicing, perhaps by affecting splicing factor recognition sites or (as depicted here) by binding to and “joining” exon regions on both sides of a particular splice junction, (**B**) affect the structural conformation of an miRNA primary transcript, determining its availability for trimming to pre-miRNA and nuclear export, (**C**) reinforce a stem loop binding motif for a localization RBP, thereby determining mRNA shuttling destination, (**D**) further lessen translation via iron regulatory protein by stabilizing a 5′ IRE in particular mRNAs, (**E**) alternatively, enhance mRNA stabilization via iron regulatory protein by stabilizing a 3′ IRE in particular mRNAs (**F**) activate structure dependent ribozymes, (**G**) act in a combinatorial fashion with multiple signals in a single mRNA.

This additional role for miRNA we have suggested could explain a variety of observed downstream effects and underscores the importance of ncRNA as a major post-transcriptional gene regulator. This model could also allow for the regulated masking or revealing of many conformational dependent mRNA regulatory elements beyond RBP binding motifs. By stabilizing or disrupting these regulatory elements, miRNAs, and possibly many other ncRNAs, could perform diverse functions with respect to individual or multiple mRNAs. In some aspects, this model is analogous to the behavior observed for riboswitches but here a miRNA is influencing shape in *trans* as opposed to a small molecule or metabolite regulating *cis* structural changes (Serganov and Nudler, 2013).

Additionally, and perhaps most biologically compelling, is the potential for nature to use sxRNA interactions to form an intricate combinatorial *trans*-acting post-transcriptional regulatory network in which various sxRNA arrangements could sequentially influence multiple RBP regulatory elements or vice versa. Analogous to the classical attenuator regulatory sequence found in some prokaryotic operons, structural changes to one or more sxRNA could then result in the conformational alterations of the structure of the mRNA such that a second RBP regulatory element can now be revealed or masked, and this could continue in a manner that would enable the post-transcriptional regulation of multi-functional genes via combinations of regulatory elements embedded in the UTRs or even the coding region of the mRNA. The sxRNA model proposed here resonates well with an earlier prosed idea of ncRNAs acting as intelligent scaffolds for the dynamic regulation of the information landscapes (Laurent et al., 2012).

## Testing the sxRNA model

Despite compelling informatic support, the sxRNA model we have proposed will need substantial supporting experimental data before it can be recognized as credible and we encourage interested researchers to participate in this process. We have successfully RIPed informatically predicted sxRNA complexes using a bacteriophage MS2-tagged-RNA construct (Yoon et al., 2012, p. 2) that contains specific HSL target sequences but although promising, these experiments can not demonstrate biological significance. Additionally, we have produced data showing that synthetic sxRNA complexes can indeed form as predicted and may prove useful as a molecular or research tool. This work is beyond the scope of this hypothesis paper but is the basis of a forthcoming technology paper.

## Author contributions

Francis Doyle and Scott A. Tenenbaum contributed equally to this manuscript.

### Conflict of interest statement

Scott A. Tenenbaum and Francis Doyle are inventors on sxRNA intellectual property that is owned by SUNY-Research Foundation and may stand to profit in accord with the SUNY-Research Foundation Patents and Inventions Policy. The SUNY-Research Foundation has entered into a licensing agreement with HocusLocus, LLC to develop and market the sxRNA technology.
